# Feasibility, Usability, and Enjoyment of a Home-Based Exercise Program Delivered via an Exercise App for Musculoskeletal Health in Community-Dwelling Older Adults: Short-term Prospective Pilot Study

**DOI:** 10.2196/21094

**Published:** 2021-01-13

**Authors:** Robin M Daly, Jenny Gianoudis, Travis Hall, Niamh L Mundell, Ralph Maddison

**Affiliations:** 1 Institute for Physical Activity and Nutrition, School of Exercise and Nutrition Sciences Deakin University Melbourne Australia

**Keywords:** home exercise, multicomponent exercise, mobile health, musculoskeletal, adherence, usability, older adults, physical activity enjoyment

## Abstract

**Background:**

Many older adults choose and prefer to exercise at home, but to attain the greatest benefits, the correct type and dose of exercise should be prescribed and adherence maintained. Advances in digital health technologies now provide the opportunity for exercise professionals to deliver and monitor personalized, evidence-based exercise programs to anyone at any time.

**Objective:**

The aim of this study was to evaluate the feasibility, usability, and enjoyment of a web-based exercise prescription app as a platform for exercise professionals to remotely deliver and monitor an individually tailored, home-based multicomponent exercise program (delivered through tablet computers) to older adults living independently in the community.

**Methods:**

This was an 8-week, prospective single-arm pilot study in 20 adults aged ≥65 years living independently in the community: 10 owned a tablet computer (tablet owners) and 10 did not own tablets (tablet nonowners). All participants were prescribed a home-based, muscle strengthening, weight-bearing impact and challenging balance/mobility program (3 days/week) using a commercial exercise prescription app on a tablet computer. Study endpoints were feasibility (retention, adherence, adverse events), usability (System Usability Scale), physical activity enjoyment (Physical Activity Enjoyment Scale), changes in lower extremity function (Short Physical Performance Battery [SPPB]), and level of physical activity (questionnaire). Process measures related to the participants' experiences and perceptions of the exercise program and web-based app were also included.

**Results:**

A total of 19 participants (mean age, 70 years) completed the study (19/20, 95%), and mean adherence to the exercise program was 84% (95% CI 70%-97%). There were 2 minor adverse events in 2 participants from 401 completed sessions. Mean weekly walking time increased by 78 minutes (95% CI 0-156, *P*=.049) and moderate-to-vigorous physical activity time by 41 minutes (95% CI –8 to 90, *P*=.09). For SPPB scores, there was a 0.3 point (95% CI –0.1 to 0.7, *P*=.17) modest sized (effect size, *d*=0.42) improvement after 8 weeks. Mean (SD) system usability was high (86 [10] with 100 best imaginable). There was no change in the overall physical activity enjoyment scores after 8 weeks, but participants reported that they enjoyed using the web-based exercise app and the exercise program (median score 4 on a 5-point Likert scale). For all measures, there were no differences between previous tablet owners and nonowners.

**Conclusions:**

This pilot feasibility study indicates that it is safe and feasible for community-dwelling older adults to participate in a home-based, multicomponent exercise program targeting musculoskeletal health and function that was delivered and monitored remotely by exercise professionals using a tablet-based exercise prescription app.

## Introduction

Every year, around 30% of people aged over 65 years living in the community fall at least once [1], and falls are a leading cause of fragility fractures, injury-related hospitalization, mortality, and health care costs for older adults [2,3]. By 2022, it is predicted that there will be 1 fragility fracture every 2.9 minutes (>500 per day) in Australia [4]. The current models of care for fracture risk reduction focus largely on pharmacological agents targeting bone mineral density (BMD) [5], but there is a need for multifaceted approaches that can simultaneously target multiple fall and fracture risk factors.

Exercise is widely recognized as a safe and effective approach to improve nearly all modifiable fracture risk factors, including BMD and falls risk [6,7]. Several meta-analyses of randomized controlled trials provide compelling evidence to support the benefits of exercise as a single intervention to prevent falls in community-dwelling older people [7] and multicomponent resistance-based exercise programs for improving bone health in postmenopausal women [8]. The findings from several of our previous randomized controlled trials conducted within community-based health and fitness centers have also shown that multicomponent exercise programs incorporating progressive resistance training combined with weight-bearing impact and challenging balance and mobility training are safe and effective for improving hip and lumbar spine BMD, muscle mass, strength, power, and function in healthy older adults and those with low BMD or at increased falls risk [9-12]. Despite these positive findings, geographical location and access to affordable community-based exercise programs and qualified exercise trainers and a general aversion to the gym environment are key barriers to participation reported by many older people, which has implications for intervention effectiveness [13-15]. Thus, there is a need to consider alternative models of service delivery to meet individuals’ exercise needs, preferences, and financial resources more broadly.

Advances in digital health apps have provided new opportunities for health care professionals to remotely deliver and monitor evidence-based exercise programs tailored to the needs of older adults and within their own home or community environment. This is important, as a study of 240 community-dwelling adults attending osteoporosis-related programs revealed that a lack of access to exercise programs that meet their needs and preferences and limited resources, time, and trust in exercise providers were some of the key barriers to participation [13]. For falls prevention, a study in 5440 older adults indicated that a home-based strength and balance training program was preferred over other prevention strategies [16]. However, to ensure clinical effectiveness, it is important that any prescribed exercise programs adhere to current best practice guidelines and incorporate behavioral strategies to promote long-term adherence. Despite the rapid rise in the number of web-based and mobile health apps available to health care professionals to deliver exercise programs to people at home, few studies have evaluated the feasibility, usability, and enjoyment of exercise prescription apps targeting the key musculoskeletal health and function (eg, bone and fall-related) risk factors associated with fractures in older adults residing in the community.

The aim of this feasibility study was to evaluate the feasibility (retention, adherence and adverse events), usability, and enjoyment of a commercial web-based exercise prescription app (Physitrack) as a platform for exercise professionals to remotely deliver and monitor an individually tailored, home-based multicomponent exercise program (via tablet computers) for older adults living independently in the community. In addition, we explored participants’ perceptions of the exercise program and Physitrack app and whether outcomes differed between previous tablet computer owners and nonowners.

## Methods

### Study Design

The Seniors Made Active thRough Technology (SMART) study was an 8-week community-based, prospective single-arm pilot study in which adults aged ≥65 years were prescribed a home exercise program (accessed by the PhysiApp-patient portal) by an accredited exercise physiologist (AEP) using the commercial Physitrack (clinician portal) exercise prescription app. The trial was managed through the Institute for Physical Activity and Nutrition at Deakin University, Australia, and was approved by the Deakin University Human Research Ethics Committee (HREC 2016-219).

### Participants and Recruitment

Twenty relatively healthy men and women (convenience sample) aged 65 years and over living independently in the community were recruited through our research trial database, and study flyers were sent to a number of community (Rotary) clubs in the eastern suburbs of Melbourne, Victoria, Australia. In order to explore differences in the outcomes and the experiences in using mobile technology between those with tablets (tablet owners) and without tablets (tablet nonowners), we deliberately recruited 10 participants who possessed a tablet computer (and had access to Wi-Fi at home) and 10 participants who did not possess such a device. The tablet nonowners were provided with an iPad and a SIM card (and adequate data capacity) for the duration of the study and instructed on how to use it during the initial home visit.

Participants were initially screened over the telephone and included if they were able to walk without the use of an aid, willing to use an iPad (their own or one provided to them) for the execution of the exercise program, and if they were able to speak English. Participants were excluded based on the following criteria (all self-reported): (1) aged <65 years, (2) participation in resistance exercise >1 session per week for at least 20 minutes or moderate-to-vigorous intensity physical activity for ≥150 min/week over the past 3 months, (3) recent low trauma fracture (within the past 6 months), (4) inability to stand unaided, (5) acute or terminal illness likely to compromise exercise participation, (6) unstable or ongoing cardiovascular/respiratory disorder, (7) musculoskeletal or neurological disease or functional limitations disrupting voluntary movement or that might have limited training, or (8) inability to commit to the study and its requirements. The Exercise and Sports Science Australia adult pre-exercise screening tool was used to identify any individual who may be at increased risk for any adverse event(s) due to participation in our exercise program. Participants with signs or symptoms of unstable or unmanaged disease were excluded from the study.

A total of 87 older adults expressed an interest and were screened for the study, of which 20 were included. The reasons for exclusion were as follows: 18 due to the presence of a cardiovascular, musculoskeletal, or neurological condition that could limit their ability to participate in the exercise program; 11 due to being too physically active; 10 due to expected travel during the study period; 3 due to age (<65 years); and 1 due to terminal illness. The other 24 participants were not interested or did not have the time to participate in the study upon receiving further details about the requirements.

### Intervention

All participants were prescribed (by a single qualified AEP recruited to work on this study) two 4-week multicomponent home exercise programs using the commercially available web-based Physitrack exercise programming app with the accompanying PhysiApp that was accessible via their iPad/tablet. Physitrack is a cloud-based, digital platform that allows health professionals to assign exercises and programs (with training dosage) to people remotely, track progress, provide feedback in real time, and send reminders. Using this program, the AEP formulated a personalized exercise program for each participant by selecting from a battery of >3500 exercises that includes narrated videos and descriptions about how to perform each exercise. The Physitrack system allows the AEP and participants to set up automated reminders about exercise times and record exercise completion, including sets, repetitions, and rate of perceived exertion (RPE) for each exercise, as well as include feedback or messages that are sent (in real time) to the AEP (or to participants from the AEP) for monitoring and review. For each exercise, participants were prescribed a specific training dose (frequency, sets, and repetitions) and asked to report on their RPE using the 10-point scale provided in the app. Each participant’s program was reviewed and progressed weekly by the AEP if needed, by reviewing the self-reported RPE and sets/repetitions for every exercise completed via the web-based Physitrack platform. The AEP also checked the Physitrack system daily for any urgent alerts/messages from participants.

Exercise prescription was individualized based on each participant’s initial functional capacity determined from the baseline assessment, medical and physical activity history, as well as the AEP’s clinical judgement. Each exercise program included a combination of muscle strengthening (resistance) exercises, weight-bearing impact activities, and challenging balance/mobility exercises based on the principles of the *Osteo-cise: Strong Bones for Life* program, which is an established and effective community-based osteoporosis prevention exercise program for older adults at increased risk for falls and fracture [11,12]. Participants were prescribed 8-9 targeted exercises (2-3 sets of 8 repetitions) at a moderate intensity (3-6 on the 10-point modified RPE scale) to be completed on 3 nonconsecutive days per week. All participants were provided with an exercise equipment pack (box step, dumbbells, TheraBand resistance bands, stepping cones, foam balance mat). The program was designed to be completed within 30 minutes and consisted of 2 warm-up exercises (eg, marching, side stepping, sit to stand), 2 challenging balance/functional exercises (eg, alternating lateral steps, tandem walking, single leg standing), 1 upper limb (eg, wall press up, triceps dips, overhead press), and 2 lower limb resistance exercises (eg, step ups, bodyweight squats, reverse lunge with weights), 2 weight-bearing impact exercises (eg, vertical, lateral, and multidirectional jumping or hopping), and cool down (stretching) activities.

All participants received 3 home visits from the AEP during the study. At the initial home visit, baseline assessments were completed and participants were educated on the PhysiApp and safe exercise training and prescribed their initial exercise program. Participants then received a weekly phone call for the first 2 weeks of the study to monitor progress and address any questions. Thereafter, participants were encouraged to liaise with the AEP directly via the PhysiApp, which was monitored daily. A second home visit was conducted at week 4 to review progress, record any adverse events, and update the exercise program, with the final home visit conducted at the end of the study (after week 8) to complete the follow-up assessments.

### Feasibility: Retention, Adherence, and Adverse Events

Retention was recorded as the number (proportion) of participants who completed the 8-week assessment. Adherence to the exercise program, including the number of sessions completed, number of exercises, and sets and repetitions completed (all expressed as a percentage) within each session were recorded within the Physitrack system. We considered the program to be feasible if at least 90% of the participants completed the trial and if the adherence to the program was at least 66% (equivalent to 2 out of 3 sessions per week). Participants were asked to record any adverse events (including falls) directly into PhysiApp so that they could be reviewed by the AEP and research staff. Information on adverse events was also collected at home visit 2 (week 4) and 3 (after week 8). An adverse event was defined as an intervention-related event resulting in absence from or modification to the exercise intervention.

### Anthropometry and Demographics

Height to the nearest 0.1 cm and body weight to the nearest 0.1 kg were measured using standard procedures. The following information was collected by the questionnaire (baseline only): date of birth, ethnic background, education, living arrangement, medical history, medication use, and history of falls. At the completion of the study, participants were also asked if they had experienced a fall(s) over the past 8 weeks.

### Physical Activity

Duration (minutes per week) of walking and moderate-to-vigorous physical activity (MVPA) during the past week, which was truncated at 840 minutes, was assessed using the validated Active Australia survey [17].

### Physical Function

Physical function was assessed using the standardized Short Physical Performance Battery (SPPB), which is a composite measure of 3 tasks: standing balance, habitual gait speed, and repeated (5) chair rise [18]. A score of 0 to 4 was assigned to each test and added to yield a composite score ranging from 0 to 12, with higher scores indicating better physical function. The SPPB has been shown to be valid, reliable, and sensitive to change with intraclass correlation coefficients of 0.88-0.92 for tests performed 1 week apart [19].

### Physical Activity Enjoyment

At baseline and follow-up, participants completed the Physical Activity Enjoyment Scale (PACES) [20]. This 18-item questionnaire asked participants to rate “*how do you feel at the moment about the physical activity you have been doing*” using a 7-point bipolar rating scale with scores ranging from 18 to 126 points. Eleven of the 18 items are reverse-scored with higher scores representing higher levels of enjoyment.

### System Usability

Usability represents the participants’ experience with using the app. At the final assessment, participants completed the System Usability Scale (SUS) [21] to assess perceived usability of Physitrack. The SUS is a standard 10-item questionnaire in which responses are measured on a 5-point Likert scale ranging from 1 (strongly disagree) to 5 (strongly agree). Questions 1, 3, 5, 7, and 9 are positive and questions 2, 4, 6, 8, and 10 are negative. A total SUS score is derived by summing the individual scores and multiplying by 2.5, which yields a score ranging between 0 (worst) and 100 (absolute best). A score >68 is considered above average usability and >80 considered high usability and a level at which participants are likely to recommend the product to peers [21].

### Process Measures: Perceptions of the Program and System

Upon trial completion, process measures were collected using an author-derived questionnaire completed by participants to evaluate their experiences with and perceptions of the home exercise program and Physitrack system. Enjoyment about the exercise program and using Physitrack was assessed on a 5-point scale (1=did not enjoy at all, 5=extremely enjoyable). In addition, participants were asked open-ended questions about what they liked and disliked most about using Physitrack. The level of importance about key elements that may have helped participants maintain their motivation to continue with the program was assessed using a 5-point scale (1=extremely important, 5=not all important). Finally, participants were asked whether they would continue to use the Physitrack app to exercise at home if it was made available. All open-ended questions were analyzed using a general inductive thematic approach [22].

### Statistical Analysis

As this was a pilot feasibility study [23], a convenience sample of 20 older adults was recruited with no formal sample size calculations [24]. However, the observed effect sizes (Cohen *d*) for the functional and physical activity measures were calculated using the following formula: mean posttest minus mean baseline divided by baseline standard deviation. The potential clinical meaningfulness of the results (in addition to statistical significance) was based on the magnitude of the effects: small (*d*=0.20), medium (*d*=0.50), and large (*d*=0.80) (and *P* values) [25]. Nevertheless, the results and findings from the hypothesis tests should be treated with caution, given our modest sample size.

All statistical analyses were conducted using SPSS Statistics for Windows, version 26 (SPSS Inc). Baseline characteristics between the group (tablet owners and nonowners) were compared using independent two-sided *t* tests for continuous variables and chi-squared tests for categorical variables. Paired sample *t* tests (two-sided) were used to assess within-group changes for the continuous variables and the McNemar test was used for categorical variables. Between-group differences for changes were assessed using analysis of variance or McNemar test for categorical variables. Between-group differences were calculated by subtracting the within-group changes from the baseline in each group. Within-group changes were presented as absolute changes from the baseline. All data were presented as mean (SD) or 95% CI (or median and interquartile range) and the significance was set at *P*<.05.

## Results

### Baseline Characteristics

The characteristics of the cohort are shown in Table 1, with no marked differences between the tablet owners and nonowners. The mean age of the 20 participants was 70 years (range 65-81 years); 50% (10/20) of the participants were females, 40% (8/20) were classified as overweight (BMI 25-29.9 kg/m^2^), and 45% (9/20) as obese (BMI ≥30 kg/m^2^), with 35% (7/20) reporting the presence of a chronic disease(s) and a median of 3 medications.

**Table 1:** Baseline characteristics of the cohort.

Baseline characteristics		Tablet owners (n=10)	Tablet nonowners (n=10)	Total (N=20)
Age (years), mean (SD)		70.1 (3.1)	70.8 (5.3)	70.4 (4.2)
Sex (% male), n (%)		5 (50)	5 (50)	10 (50)
Height (cm), mean (SD)		165.1 (12.4)	165.7 (9.3)	165.4 (10.7)
Weight (kg), mean (SD)		83.5 (14.3)	81.5 (20.2)	82.5 (17.1)
BMI (kg/m^2^), mean (SD)		30.7 (5.0)	29.3 (5.1)	30.0 (5.0)
**Ethnicity, n (%)**				
	Caucasian	6 (60)	3 (30)	9 (45)
	Other	4 (40)	7 (70)	11 (55)
**Highest level of education, n (%)**				
	Primary/High school	3 (30)	0 (0)	3 (15)
	University or Tertiary level	7 (70)	5 (50)	12 (60)
	Technical/Trade certificate	0 (0)	5 (50)	5 (25)
**Living arrangement, n (%)**				
	Alone	0 (0)	3 (30)	3 (15)
	With adult without children	9 (90)	5 (50)	14 (70)
	With adult with children	0 (0)	1 (10)	1 (5)
	Retirement village/hostel	1 (10)	1 (10)	2 (10)
**Marital status, n (%)**				
	Married/De Facto	9 (90)	8 (80)	17 (85)
	Separated/Divorced/Widowed	1 (10)	2 (20)	3 (15)
Number of medications, median (IQR)		3.5 (1.0-4.0)	2.5 (1.0-5.5)	3.0 (1.0-4.0)
Presence of chronic disease(s),^a^ n (%)		5 (50)	2 (20)	7 (35)
Previous fall in past 12 months, n (%)		2 (10)	0 (0)	2 (10)

^a^Presence of chronic disease include self-reported hypertension, cardiovascular disease, stroke, Alzheimer disease, Parkinson disease, chronic kidney disease, liver disease, type 2 diabetes, or a neurological/brain disease.

### Feasibility: Retention, Adherence, and Adverse Events

Study retention was 95% (19 of 20 participants completed the study). Mean exercise adherence over the 8 weeks was 84% (95% CI 70%-97%, median 94%) and was no different between tablet owners and nonowners (mean 95% vs 72%, *P*=.07). Mean adherence to the prescribed number of exercises per session was 81% (95% CI 68%-95%), number of sets was 82% (95% CI 68%-96%), and the number of repetitions for each exercise was 81% (95% CI 68%-95%), with no differences between the 2 groups (*P*>.05). Over the 8-week program, 1 musculoskeletal complaint (knee pain that was pre-existing) and 1 injury (strained calf muscle) was reported by 2 participants. The participant with knee pain continued to exercise with a modified program, while the second participant sought treatment and subsequently withdrew from the study. No falls were reported by any participant over the 8-week study. A total of 72 in-app messages (from 480 prescribed exercise sessions) were sent to the AEP by 9 of the 20 participants (median 6 per person).

### Physical Activity

In the total cohort, mean weekly time spent walking increased on average by 78 minutes (95% CI 0-156, *P*=.049; *d*=0.66] (Table 2). Thirteen participants (68%) reported an increase in the weekly walking time (range 10-360 minutes), 2 (11%) reported no change, and 4 (21%) reported a decrease (range 30-240 minutes). For MVPA, there was a mean change of 41 minutes (95% CI –8 to 90, *P*=.09; *d*=0.35]. Nine participants (47%) reported an increase in the weekly MVPA time (range 20-240 minutes), 6 (32%) reported no change, and 4 (21%) reported a decrease (range 15-180 minutes). There were no significant between-group differences (tablet versus not tablet owners) for the change in either physical activity variable.

**Table 2:** Changes in physical activity and the Short Physical Performance Battery scores for the tablet owners, tablet nonowners, and all participants combined.

Parameters		Baseline (N=20), mean (SD)	Week 8 (n=19), mean (SD)	Mean change (95% CI)	Effect size (Cohen *d*)
**Walking time (min/week)**					
	Tablet owners	154 (84)	225 (155)	67 (–32 to 166)	1.20
	Tablet nonowners	155 (154)	244 (251)	89 (–50 to 227)	0.58
	All	155 (121)	235 (206)	78 (0 to 156)^a^	0.66
**Moderate-to-vigorous physical activity (min/week)**					
	Tablet owners	50 (88)	97 (164)	42 (–37 to 121)	0.54
	Tablet nonowners	151 (152)	191 (217)	40 (–36 to 116)	0.26
	All	100 (132)	147 (195)	41 (–8 to 90)	0.36
**Short Physical Performance Battery score**					
	Tablet owners	11.3 (0.7)	11.7 (0.7)	0.3 (–0.3 to 1.0)	0.59
	Tablet nonowners	11.7 (0.7)	11.9 (0.3)	0.2 (–0.4 to 0.8)	0.30
	All	11.5 (0.7)	11.8 (0.5)	0.3 (–0.1 to 0.7)	0.42

^a^*P*=.049 within group change after 8 weeks.

### Physical Function

After 8 weeks, there was a nonsignificant mean 0.3 point (95% CI –0.1 to 0.7, *P*=.17) improvement in the composite SPPB score in all participants, which represented a moderate effect (*d*=0.42) (Table 2). Five participants (26%) had an improvement of one or more points in SPPB performance, 12 (63%) had no change, and 2 (11%) experienced a reduction. There were no group differences for the change in the mean composite SPPB scores.

### SUS

Based on SUS, the Physitrack app was reported to be highly usable by all participants (mean score 86, SD 10), with no group differences (Table 3). For the 10 individual questions, participants most strongly agreed that they felt confident using it and that most people would learn to use the system very quickly. Participants also strongly disagreed that the system was cumbersome and unnecessarily complex.

**Table 3:** Means and standard deviation scores for the System Usability Scale.

Questions^a^	Tablet owners	Tablet nonowners	All
I think I would like to use the app frequently	4.22 (0.67)	4.00 (1.05)	4.11 (0.88)
I found the system to be unnecessarily complex	1.33 (0.50)	1.40 (0.52)	1.37 (0.50)
I thought the system was easy to use	4.22 (1.30)	4.40 (0.97)	4.32 (1.11)
I think that I would need support of a technical person to be able to use the system	1.67 (0.87)	1.60 (0.84)	1.63 (0.83)
I found the various functions in the system were well integrated	4.22 (0.44)	4.10 (0.99)	4.16 (0.77)
I thought there was too much inconsistency in the system	2.00 (1.10)	1.50 (0.53)	1.74 (0.81)
I would imagine that most people would learn to use the system very quickly	4.56 (0.53)	4.30 (0.68)	4.42 (0.61)
I found the system very cumbersome to use	1.11 (0.33)	1.30 (0.48)	1.21 (0.42)
I felt very confident using the system	4.67 (0.50)	4.80 (0.42)	4.74 (0.45)
I needed to learn a lot of things before I could get going with the system	1.56 (0.73)	1.60 (1.27)	1.58 (1.02)
System Usability Scale total score (out of 100)	85.6 (7.6)	85.5 (11.8)	85.5 (9.8)

^a^Responses were scored on a 5-point Likert scale: 1=strongly disagree, 5=strongly agree.

### PACES

In all participants, the mean (SD) physical activity enjoyment (PACES) scores did not change over time (baseline 71.2 [8.6] vs 8 weeks 69.2 [7.9], *P*=.45) nor differ between the groups (*P*=.07).

### Process Measures: Perceptions of the Program and System

Participants reported that they enjoyed using the Physitrack app and participating in the exercise program (median score 4 out of 5), with ease of use and the narrated video demonstrations of the exercises within the app reported by participants as to what they liked most. Overall, 71% (12/17) of the participants reported no response in terms of what they disliked most about the app but a lack of clarity around the terminology (eg, the terms reps and sets) was reported by 3 participants (17%). Participants’ own motivation rather than the use of the app and interactions with the exercise trainer or potential health improvements were rated as the most important factor for them continuing with the program over the 8 weeks. Finally, 94% (16/17) of the participants reported that they would recommend the home-based program to other older people and 88% (15/17) would continue to use the Physitrack app and exercise at home if it were made available (Table 4).

**Table 4:** Participants’ perceptions of the exercise program and web-based Physitrack exercise programming system (n=17).

Questions		Scores
How did you enjoy participating in the home-based exercise program?^a^, median=4.0, mean (SD)		3.50 (0.97)
How did you enjoy using the Physitrack online exercise system?^a^, median=4.0, mean (SD)		3.88 (0.86)
**What did you like most about the Physitrack system (app)? n (%)**		
	Ease of use	7 (41)
	Narrated video demonstrations of exercises	7 (41)
	Ability to track and modify program, if needed	3 (18)
**What did you like least about the Physitrack system (app)? n (%)**		
	Lack of clarity around terminology	3 (17)
	Lack of flexibility if exercising on a different day	1 (6)
	Just another piece of equipment (device) to use when exercising	1 (6)
	No response	12 (71)
**How important was each of the following to help you maintain your motivation to continue with the program?^b^, median=2.0, mean (SD)**		
	Own motivation	1.88 (0.93)
	Physitrack system	2.18 (1.01)
	Exercise trainer	2.24 (1.03)
	Feedback from trainer and performance assessments	2.24 (1.03)
	Improvements to health	2.47 (1.07)
Do you feel that taking part in the program improved your health and fitness? n (%)		15 (88)
Would you recommend the home-based program to other older people? n (%)		16 (94)
If the Physitrack system continued to be available, would you continue to exercise at home? n (%)		15 (88)

^a^Responses scored on a 5-point scale: 1=did not enjoy at all, 5=extremely enjoyable.

^b^Responses scored on a 5-point scale: 1=extremely important, 5=not important at all.

## Discussion

### Summary of the Main Findings

Overall, the findings from our SMART prospective pilot feasibility study indicate that it was safe and feasible for exercise professionals to prescribe and remotely monitor a thrice weekly home-based, multicomponent exercise program targeting musculoskeletal health and function delivered via the Physitrack app for older adults living independently in the community. This feasibility was evident by the low attrition, high adherence to the exercise training, low number of adverse events, increased weekly physical activity time, and the high reported usability. No differences were observed in the outcomes between participants who were previous owners and nonowners of a tablet computer. Participants reported that they enjoyed using the Physitrack app and participating in the exercise program. Most participants stated that they would recommend the home-based exercise program to other older people and continue to use the exercise programming app if it was still available, which adds further support to the widespread usability and acceptability of our intervention and the web-based exercise programming app.

### Comparison With Prior Work

Consistent with our findings, several previous interventions conducted over 2-6 months have reported that the use of web/mobile exercise prescription apps represent a safe, effective, and feasible approach to deliver tablet-based, home muscle strength and balance training for older people [26-30]. However, there were marked differences in the attrition rates (8%-47%) in some of these studies, and adherence to the exercise training ranged from 61% to 73% [27-29]. This heterogeneity is likely related to factors such as whether additional behavioral change strategies or remote support were provided or not, differences in the stability of internet connections, and duration of the interventions. In our 8-week study, the low attrition and high exercise adherence are likely due to several components related to both the intervention and web-based exercise programming system. This includes the prescription of individualized exercise programs based on individual’s functional status and health/medical history, the initial telephone calls and home visits by the AEP, the option for participants to communicate with the AEP via the app at any time to receive feedback/support, knowing that the AEP was remotely monitoring all exercise programs, and the shorter study duration. Further data to support the feasibility and usability of the exercise programming system used in our study is highlighted by the findings from a 3-week pragmatic randomized controlled trial in 305 adults being treated for a musculoskeletal condition(s) [31]. In this study, it was found that the use of the Physitrack system by physical therapists improved home exercise adherence and confidence in the ability of patients to undertake exercise at home compared to usual care (eg, written exercise instructions, printed exercise diagrams) [31].

Although our study was not designed nor powered to detect an effect of the intervention on physical function, we did observe a modest effect (*d*=0.42) on improving SPPB scores (mean change 0.3 points). Previous research has indicated that a change in SPPB of 0.3-0.8 points represents a minimally important (significant) change [32], with a change for 0.5 and 1.0 point classified as a small but meaningful change and substantial change, respectively [33]. In our study, it is likely that the modest changes relate to the initial functional status of our participants, who had a mean SPPB score of 11.5 out of 12. Indeed, this may explain why only 26% (n=5) of the participants experienced an improvement of one or more points on the SPPB test after 8 weeks. Nevertheless, our findings must be interpreted with caution given that there was no control (nonexercise) comparison group. For comparison, a previous 12-week tablet-based, home strength and balance training program in 44 independently living older adults found that the intervention and control groups experienced similar significant improvements in SPPB scores [26].

An important finding from our study was that participants were enthusiastic about the web-based Physitrack system and its ease of use, independent of whether they had previously owned a tablet computer and would be willing to continue to use such a system in the future. Despite these positive experiences, overall enjoyment in physical activity did not change over the 8 weeks, which may be related to the relatively short duration of the exercise program or that the questionnaire we used to monitor physical activity enjoyment may not measure the ideal constructs specific to the intervention. The lack of any marked changes in physical activity enjoyment (and SPPB performance) may also be due in part to the fact that participants recruited into the study were already habitually active (mean, 100 minutes of MVPA per week). While future studies are needed to evaluate the long-term acceptability, adherence, and clinical effectiveness of remotely prescribed web-based exercise programs using apps for older adults, we wish to highlight that we are not recommending that such approaches replace traditional community-based exercise programs but provide an alternative option that might best meet some individuals’ needs, preferences, and financial resources.

An interesting observation from our study was the increase in weekly walking time following the 8-week home-based, exercise program. It is important to note that participants were not specifically instructed to engage in any additional physical activity outside of the intervention. Thus, the reason(s) for the mean 78 minutes per week increase in weekly walking time is difficult to explain. However, there is some evidence that participation in structured exercise programs is associated with an increase in nonprescribed activity and energy expenditure outside of the intervention [34]. It has been suggested that this may be related to a number of factors, including exercise-related improvements in functional capacity, gains in muscle strength, reduced levels of fatigue or feeling more energetic, improvements in exercise self-efficacy, or mood [34]. However, others have observed that adoption of structured exercise leads to no change or a decrease in habitual physical activity or energy expenditure, which has been attributed to some compensatory behavioral adaptation [35]. Given the small sample size and wide confidence interval for the change in mean weekly walking time in our study, these findings must be interpreted with caution but warrant further follow-up to understand the reason(s) why home-based exercise training may improve habitual activity levels.

### Limitations

This study has a number of limitations, including the small sample size, convenience sample that limits generalizability, pretest-posttest study design, relatively short intervention duration, lack of blinding of the assessor, lack of a nonexercise control group, the use of self-reported measures of physical activity, and the inclusion of generally healthy and physically active older adults with normal functional capacity, which may also affect generalizability. The 3 home-based visits by the AEP to brief participants on the use of the app and exercise program and to conduct the functional tests is a further limitation in terms of future widespread scalability. The need for the AEP to regularly review and monitor the messages/alerts from participants and their weekly progress using the Physitrack app/platform could be considered burdensome, but the daily time commitment was typically less than 5 minutes. Finally, the addition of semistructured interviews may provide further insights into the experiences and perspectives of the participants, including potential differences between tablet computer owners and nonowners.

### Conclusion

This pilot feasibility study indicates that it was safe and feasible for older adults living independently in the community to participate in a tablet-computer–delivered, home-based, multicomponent exercise program targeting musculoskeletal health and function that was developed and monitored remotely by exercise professionals using a web-based exercise prescription app.

## Abbreviations

AEP: accredited exercise physiologist
BMD: bone mineral density
MVPA: moderate-to-vigorous physical activity
PACES: physical activity enjoyment scale
RPE: rate of perceived exertion
SMART: Seniors Made Active thRough Technology
SPPB: short physical performance battery
SUS: system usability scale

## References

[1] Bergen G, Stevens MR, Burns ER (2016). Falls and Fall Injuries Among Adults Aged ≥65 Years - United States, 2014. MMWR Morb Mortal Wkly Rep.

[2] AIHW (2012). Hospitalisations due to falls by older people, Australia 2006-07. Injury research and statistics series no. 57. Cat. No. INJCAT 133. Canberra, AIHW.

[3] AIHW (2017). Trends in hospitalisations due to falls by older people, Australia 2002-03 to 2012-13. Injury research and statistics series no. 106. Cat. no. INJCAT 182. Canberra, AIHW.

[4] Watts JJ, Abimanyi-Ochom J, Sanders K (2013). Osteoporosis costing all Australians: A new burden of disease analysis-2012 to 2022, Glebe, NSW: Osteoporosis Australia.

[5] Eastell R, Rosen CJ, Black DM, Cheung AM, Murad MH, Shoback D (2019). Pharmacological Management of Osteoporosis in Postmenopausal Women: An Endocrine Society* Clinical Practice Guideline. J Clin Endocrinol Metab.

[6] Daly RM, Dalla Via J, Duckham RL, Fraser SF, Helge EW (2019). Exercise for the prevention of osteoporosis in postmenopausal women: an evidence-based guide to the optimal prescription. Braz J Phys Ther.

[7] Sherrington C, Fairhall N, Wallbank G, Tiedemann A, Michaleff ZA, Howard K, Clemson L, Hopewell S, Lamb S (2020). Exercise for preventing falls in older people living in the community: an abridged Cochrane systematic review. Br J Sports Med.

[8] Zhao R, Zhao M, Xu Z (2015). The effects of differing resistance training modes on the preservation of bone mineral density in postmenopausal women: a meta-analysis. Osteoporos Int.

[9] Kukuljan S, Nowson CA, Sanders KM, Nicholson GC, Seibel MJ, Salmon J, Daly RM (2011). Independent and combined effects of calcium-vitamin D3 and exercise on bone structure and strength in older men: an 18-month factorial design randomized controlled trial. J Clin Endocrinol Metab.

[10] Kukuljan S, Nowson CA, Sanders K, Daly RM (2009). Effects of resistance exercise and fortified milk on skeletal muscle mass, muscle size, and functional performance in middle-aged and older men: an 18-mo randomized controlled trial. J Appl Physiol (1985).

[11] Gianoudis J, Bailey CA, Ebeling PR, Nowson CA, Sanders KM, Hill K, Daly RM (2014). Effects of a targeted multimodal exercise program incorporating high-speed power training on falls and fracture risk factors in older adults: a community-based randomized controlled trial. J Bone Miner Res.

[12] Daly RM, Gianoudis J, Kersh ME, Bailey CA, Ebeling PR, Krug R, Nowson CA, Hill K, Sanders KM (2020). Effects of a 12-Month Supervised, Community-Based, Multimodal Exercise Program Followed by a 6-Month Research-to-Practice Transition on Bone Mineral Density, Trabecular Microarchitecture, and Physical Function in Older Adults: A Randomized Controlled Trial. J Bone Miner Res.

[13] Ziebart C, McArthur C, Lee L, Papaioannou A, Laprade J, Cheung AM, Jain R, Giangregorio L (2018). "Left to my own devices, I don't know": using theory and patient-reported barriers to move from physical activity recommendations to practice. Osteoporos Int.

[14] Cavill NA, Foster CE (2018). Enablers and barriers to older people’s participation in strength and balance activities: A review of reviews. JFSF.

[15] Timmons JF, Griffin C, Cogan KE, Matthews J, Egan B (2020). Exercise Maintenance in Older Adults 1 Year After Completion of a Supervised Training Intervention. J Am Geriatr Soc.

[16] Yardley L, Kirby S, Ben-Shlomo Y, Gilbert R, Whitehead S, Todd C (2008). How likely are older people to take up different falls prevention activities?. Prev Med.

[17] Australian Institute of Health and Welfare (2003). The Active Australia Survey: A guide and manual for implementation, analysis and reporting.

[18] Guralnik JM, Ferrucci L, Pieper CF, Leveille SG, Markides KS, Ostir GV, Studenski S, Berkman LF, Wallace RB (2000). Lower extremity function and subsequent disability: consistency across studies, predictive models, and value of gait speed alone compared with the short physical performance battery. J Gerontol A Biol Sci Med Sci.

[19] Ostir GV, Volpato S, Fried LP, Chaves P, Guralnik JM (2002). Reliability and sensitivity to change assessed for a summary measure of lower body function. Journal of Clinical Epidemiology.

[20] Kendzierski D, DeCarlo K (1991). Physical activity enjoyment scale: two validation studies. Int J Sport Exerc Psychol.

[21] Bangor A, Kortum PT, Miller JT (2008). An Empirical Evaluation of the System Usability Scale. International Journal of Human-Computer Interaction.

[22] Braun V, Clarke V (2006). Using thematic analysis in psychology. Qualitative Research in Psychology.

[23] Abbade LPF, Abbade JF, Thabane L (2018). Introducing the CONSORT extension to pilot trials: enhancing the design, conduct and reporting of pilot or feasibility trials. J Venom Anim Toxins Incl Trop Dis.

[24] Whitehead AL, Julious SA, Cooper CL, Campbell MJ (2016). Estimating the sample size for a pilot randomised trial to minimise the overall trial sample size for the external pilot and main trial for a continuous outcome variable. Stat Methods Med Res.

[25] Cohen J (1988). Statistical power analysis for the behavioral sciences.

[26] van Het Reve Eva, Silveira P, Daniel F, Casati F, de Bruin ED (2014). Tablet-based strength-balance training to motivate and improve adherence to exercise in independently living older people: part 2 of a phase II preclinical exploratory trial. J Med Internet Res.

[27] Silveira P, van de Langenberg Rolf, van Het Reve Eva, Daniel F, Casati F, de Bruin Eling D (2013). Tablet-based strength-balance training to motivate and improve adherence to exercise in independently living older people: a phase II preclinical exploratory trial. J Med Internet Res.

[28] Geraedts HAE, Zijlstra W, Zhang W, Spoorenberg SLW, Báez Marcos, Far IK, Baldus H, Stevens M (2017). A Home-Based Exercise Program Driven by Tablet Application and Mobility Monitoring for Frail Older Adults: Feasibility and Practical Implications. Prev Chronic Dis.

[29] Nikitina S, Didino D, Baez M, Casati F (2018). Feasibility of Virtual Tablet-Based Group Exercise Among Older Adults in Siberia: Findings From Two Pilot Trials. JMIR Mhealth Uhealth.

[30] Hong J, Kong H, Yoon H (2018). Web-Based Telepresence Exercise Program for Community-Dwelling Elderly Women With a High Risk of Falling: Randomized Controlled Trial. JMIR Mhealth Uhealth.

[31] Bennell KL, Marshall CJ, Dobson F, Kasza J, Lonsdale C, Hinman RS (2019). Does a Web-Based Exercise Programming System Improve Home Exercise Adherence for People With Musculoskeletal Conditions?: A Randomized Controlled Trial. Am J Phys Med Rehabil.

[32] Kwon S, Perera S, Pahor M, Katula JA, King AC, Groessl EJ, Studenski SA (2009). What is a meaningful change in physical performance? Findings from a clinical trial in older adults (the LIFE-P study). J Nutr Health Aging.

[33] Perera S, Mody S, Woodman R, Studenski S (2006). Meaningful change and responsiveness in common physical performance measures in older adults. J Am Geriatr Soc.

[34] Drenowatz C, Grieve GL, DeMello MM (2015). Change in energy expenditure and physical activity in response to aerobic and resistance exercise programs. Springerplus.

[35] Melanson EL (2017). The effect of exercise on non-exercise physical activity and sedentary behavior in adults. Obes Rev.

